# Unraveling variations of crystalline cellulose induced by ionic liquid and their effects on enzymatic hydrolysis

**DOI:** 10.1038/s41598-017-09885-9

**Published:** 2017-08-31

**Authors:** Zhe Ling, Sheng Chen, Xueming Zhang, Keiji Takabe, Feng Xu

**Affiliations:** 1 Beijing Key Laboratory of Lignocellulosic Chemistry, Beijing Forestry University, Beijing, 100083 China; 2 Laboratory of Tree Cell Biology, Graduate School of Agriculture, Kyoto University, Kyoto, 606-8502 Japan

## Abstract

Ionic liquid (IL) is one of the pretreatment processes gaining considerable interests to remove the native recalcitrance of lignocellulose. But the cellulose crystalline transformation during the pretreatment and their correlations with enzymatic digestibility have not been fully elucidated. Microcrystalline cellulose (Avicel) and holocellulose, which have differential sources and original crystallinity, were respectively pretreated with 1-butyl-3-methylimidazolium chloride ([C4min]Cl). Cellulose crystalline variations as well as chemical and morphological changes were determined. Crystallinity of different materials was proved to influence the effects of pretreatment and following enzymatic digestibility. Recrystallized cellulose Iβ was revealed from slight initial cellulose Iα of Avicel, which was accomplished via formation of intermediate paracrystalline phases. The conversion yield of IL pretreated Avicel displayed no obvious changes, mainly resulted from initial high crystalline order and the recrystallization behavior. Recalcitrance of holocellulose was destroyed during cellulose allomorph transformation and hemicelluloses extraction, contributing to significant increase of glucose yield up to 92.20%. Explicit comprehension on cellulose supramolecular structure may help provide more efficient process for bioconversion after IL pretreatment.

## Introduction

Ever growing demands for global energy as well as the concerns on climate change have directed researchers to pursue alternative renewable sources for the sustainable production of fuels^1^. Lignocellulose biomass, initially composed of cellulose, hemicelluloses and lignin, is a renewable and widely available raw material for bioconversion to fuels and value-added materials^2^. Cellulose is the main component of lignocellulose and combined in excellent form with lignin and hemicelluloses, making up a unique high-strength and durable material. It has become the most abundant natural carbohydrate resource on the earth^3^.

Cellulose is a high molecular weight polysaccharide consisting of D-glucopyranose units linked by β-1, 4-glycosidic bonds^4^. The intra- and intersheet hydrogen bonding between hydroxyl groups and oxygens result in the linear configuration of the cellulose chains, which stack into elementary fibrils and further aggregate into larger microfibrils^5^. Within the cellulose fibrils, there are regions where the cellulose chains are arranged in a highly ordered (crystalline) structure and regions that are disordered (amorphous)^6^. Pure cellulose exists in several crystalline allomorphs with different packing arrangements^7^. Native cellulose I, commonly derived from a variety of organisms (plants, tunicates, algae and bacteria), is recognized to crystallize in a one-chain triclinic structure Iα and a two-chain monoclinic Iβ form. Both allomorphs are packed in a parallel chain arrangement, but have various ratios in a fiber, depending on the origin^8^. Cellulose Iα is the dominate polymorph for algae and bacteria, whereas cellulose Iβ is primarily from higher plant cell wall and tunicates. The conversion from Iα to Iβ can be accomplished by annealing treatment at high temperature (~260 °C)^9^. The conversion is controlled by adjusting the treating parameters, while few reports realized complete transformation from Iα to Iβ, the more stable polymorph^10^. Cellulose II is prepared by either mercerization or regeneration from cellulose I and it crystallizes in monoclinic anti-parallel chains^11^. Mercerization involves intracrystalline swelling of cellulose in concentrated aqueous alkali followed by washing and recrystallization. Regeneration refers to either preparing a cellulose solution in an appropriate solvent or making an intermediate derivative followed by coagulation and recrystallization^12^. Several other cellulose allomorphs have also been researched in recent years^13^. Differences in glucan chain packing in these polymorphs have been shown to influence the cellulose conversion to monosaccharides^14^.

Composed of crystalline cellulose nanofibrils embedded in an amorphous matrix of cross-linked lignin and hemicelluloses, lignocellulose shows the natural recalcitrance that impedes enzyme and microbial accessibility^15^. Thus, lignocellulose biomass must be pretreated prior to addition of hydrolytic enzymes for saccharification of cellulose, as yields are otherwise too low^16^. Ionic Liquid (IL) has recently been developed as promising and powerful green solvents for pretreatment and dissolution of biomass. Dissolved and regenerated from IL, the salts with melting point at or below 100 °C, cellulose structure is capable to be changed by disrupting crystalline orders or breaking chemical linkages^17^. For pretreated biomass, the effect of IL can be summed up as: decrystallization or crystalline transformation from cellulose I to cellulose II; partial reduction of cellulose degree of polymerization and extraction of hemicelluloses and lignin matrix which can also be summarized as the disruption of lignin-carbohydrate complex (LCC)^18^. As a result, IL pretreatment was proved to be an ideal method for enhancing enzymatic conversion to monosaccharides^19^.

It has been commonly believed that the original cellulose I converted to amorphous structure or cellulose II allomorph during IL pretreatment, which depends on treating condition and types of biomass or ionic liquids (ILs). The process of IL pretreatment suggested disorder of parallel stacking, expansion of crystal lattice and smoother surface of cellulose II, which showed higher enzymatic digestibility than cellulose I, especially when hydrated^20^. Inner crystal changes were also reported by other researchers. Samayam *et al*. firstly proposed that cellulose in corn stover and switchgrass recrystallized back to cellulose I when incubated in 1-ethyl-3-methylimidazolium acetate ([C2mim][OAc])^17^. Similar results were obtained at the same year by Lucas *et al*. on poplar researches^21^. They reported that recrystallization to cellulose I within the IL intercalated fibers was detected with generation of an intermediate structure. To sum up, [C2mim][OAc] pretreatment on biomass materials at relatively low temperature (50–60 °C) caused apparent performance on recrystallization to cellulose I. However, the phenomenon was rarely reported for other categories of IL or on pure cellulose samples, even though the mixture of remnant cellulose I and regenerated cellulose II was found by Kim *et al*.^22^. Furthermore, detailed understandings on relationship between cellulose crystalline transformation and enzymatic digestibility have not been fully elucidated.

Here, we chose artificial microcrystalline cellulose (Avicel) as highly crystallized pure cellulose material and lignin-free holocellulose of moso bamboo (*Phyllostachys edulis*) as biomass derived cellulose material. With similar cellulose crystalline allomorph but different initial crystallinity, the materials were pretreated by a kind of environmentally friendly and inexpensive IL 1-n-butyl-3-methylimidazolium chloride ([C4mim]Cl) for varying pretreatment time. Chemical compositional and morphological changes of untreated and pretreated samples were analysed. Cellulose structural characterizations such as crystallinity (CrI), crystallite size, lattice spacings as well as the microfibril arrangements were determined quantitatively. Sufficient comprehension of cellulose crystalline alternations for different cellulosic materials was obtained, which will be beneficial for interpreting the mechanisms about enhanced enzymatic hydrolysis after IL pretreatment.

## Results and Discussion

### Chemical composition

The chemical changes of Avicel and holocellulose samples were determined by compositional analysis (Table 1). As can be seen, untreated Avicel (A0) represented the cellulose content of 96.54%, extremely close to the pure cellulose. With the rising time of [C4mim]Cl pretreatment, the content of cellulose experienced very slight decrease. About 91% of cellulose remained in 10 h pretreated Avicel (A10). This phenomenon indicated that, despite of some mass loss during the dissolution and regeneration process, the cellulose molecular structures stayed unchanged in regenerated Avicel samples. Lignin-free bamboo holocellulose (H0) contained 59.40% of glucose, indicating that cellulose acts as the main component. The hemicelluloses of native sample accounted for 32.22%, which consist of xylose (26.51%) and arabinose (5.71%), reflecting the main polysaccharide in bamboo hemicelluloses, arabinoxylan. The IL pretreatment on holocellulose initially caused the degradation of hemicelluloses, which was gradual with prolonging reaction time. The content of hemicelluloses decreased to 19.32% for H1 and 17.52% for H4 respectively. The severe pretreatment condition (10 h) resulted in further loss of hemicelluloses to 12.81% of H10, ascending the relative content of glucose to 83.34% simultaneously. Xylose, which decreased from 26.51% (H0) to 9.90% (H10) made the main contribution for hemicelluloses degradation. Progressive extraction and dissolution of lignin and hemicelluloses matrix by breaking the major chemical linkages of various feedstocks have been discussed previously^23^. Removing the effect of lignin for IL pretreatment, holocellulose used in the current work distinctly proved the dissolution of hemicelluloses, in which xylose was the main composition sensitive to ILs.

**Table 1:** Chemical composition analysis of Avicel and holocellulose samples before and after [C4mim]Cl pretreatment.

Samples	Glucose (%)	Xylose (%)	Arabinose (%)	Galactose (%)	Total Hemicelluloses (%)
A0	96.54 ± 1.13	—	—	—	—
A1	94.87 ± 2.60	—	—	—	—
A4	94.06 ± 2.77	—	—	—	—
A10	90.55 ± 3.04	—	—	—	—
H0	59.40 ± 1.64	26.51 ± 0.24	5.71 ± 0.10	—	32.22 ± 0.34
H1	64.53 ± 0.78	16.07 ± 0.81	3.11 ± 0.09	0.14 ± 0.02	19.32 ± 0.92
H4	76.19 ± 1.73	14.56 ± 0.65	2.83 ± 0.24	0.13 ± 0.01	17.52 ± 0.70
H10	83.34 ± 2.52	9.90 ± 0.58	2.70 ± 0.17	0.21 ± 0.02	12.81 ± 0.77

**Figure 1:**
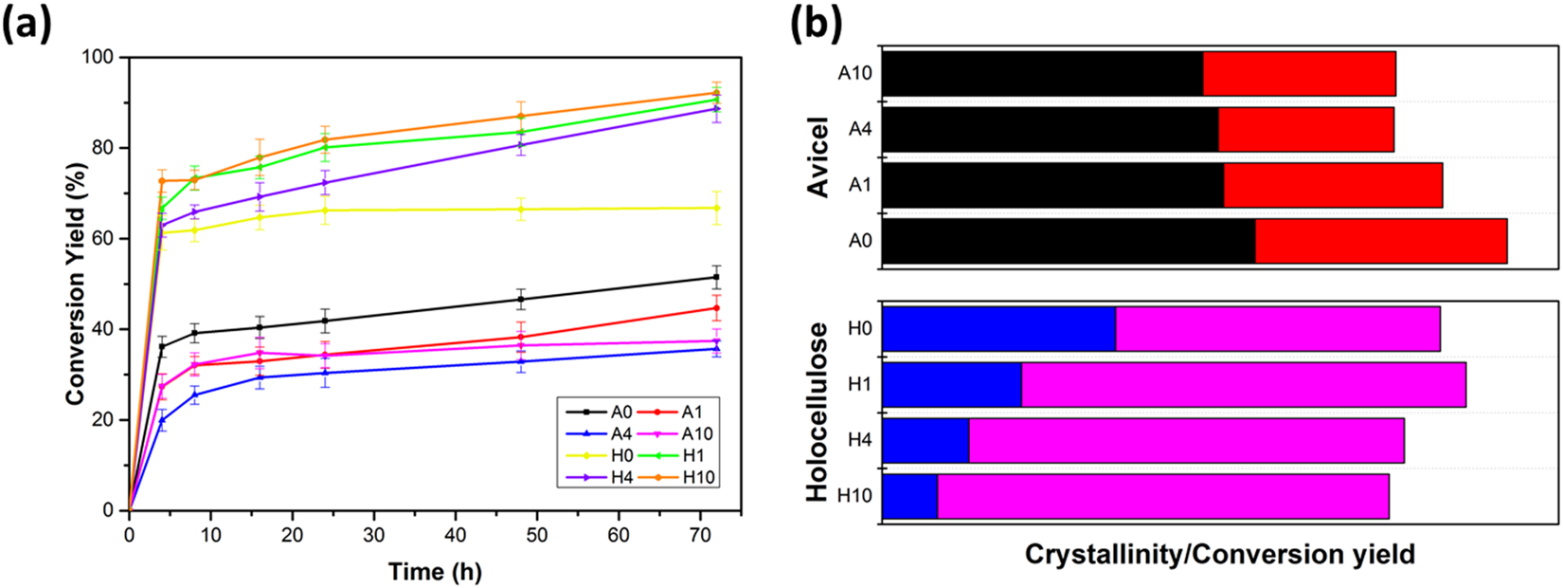
FTIR spectra of (**a**) Avicel and (**b**) holocellulose samples pretreated by [C4mim]Cl with different incubation time.

Fourier transform infrared spectroscopy (FTIR) analysis provided more specific information of Avicel and holocellulose chemical composition before and after the IL pretreatment (Fig. 1). The spectral region of FTIR was 4000 cm^−1^–700 cm^−1^. For Avicel samples, the absorbance at 3400 cm^−1^ assigned to –OH stretching vibration of cellulose showed no obvious changes, indicating the high yield of cellulose in regenerated Avicel. The conclusion can also be inferred from the similar peaks at 2900 cm^−1^ and 895 cm^−1^ respectively attributed to C-H stretching and β-glycosidic linkages of cellulose during the IL pretreatment^24^. The FTIR results on Avicel are consistent with compositional analysis.

FTIR spectra of holocellulose provided more apparent changes of chemical linkages during IL pretreatment compared to that of Avicel. The peak at 1736 cm^−1^ and 1637 cm^−1^ are respectively typical of stretching vibration in acetyl groups and OH bending vibration of hydration water in xylan-type polysaccharides. These two peaks seemed to be sharper with rising incubation time, revealing the disruption of inner linkages of hemicelluloses, especially the xylan^25^. Small absorbance at 1040 cm^−1^ associated with arabinoxylans of hemicelluloses is visible on untreated holocellulose spectrum. The peak decreased with rising pretreatment time, and was almost disappeared for 10 h pretreated holocellulose. It is said that the extraction of hemicelluloses in chlorides IL is not so potent as sulfonate or acetate ILs, whereas the hemicelluloses removal is remarkable in this work^26^. Delignification before IL pretreatment that broke the LCC of raw material, may play an important role in considerable removal of hemicelluloses, converting into furfurals or humins^27^.

### Morphological analysis of cellulose samples

**Figure 2:**
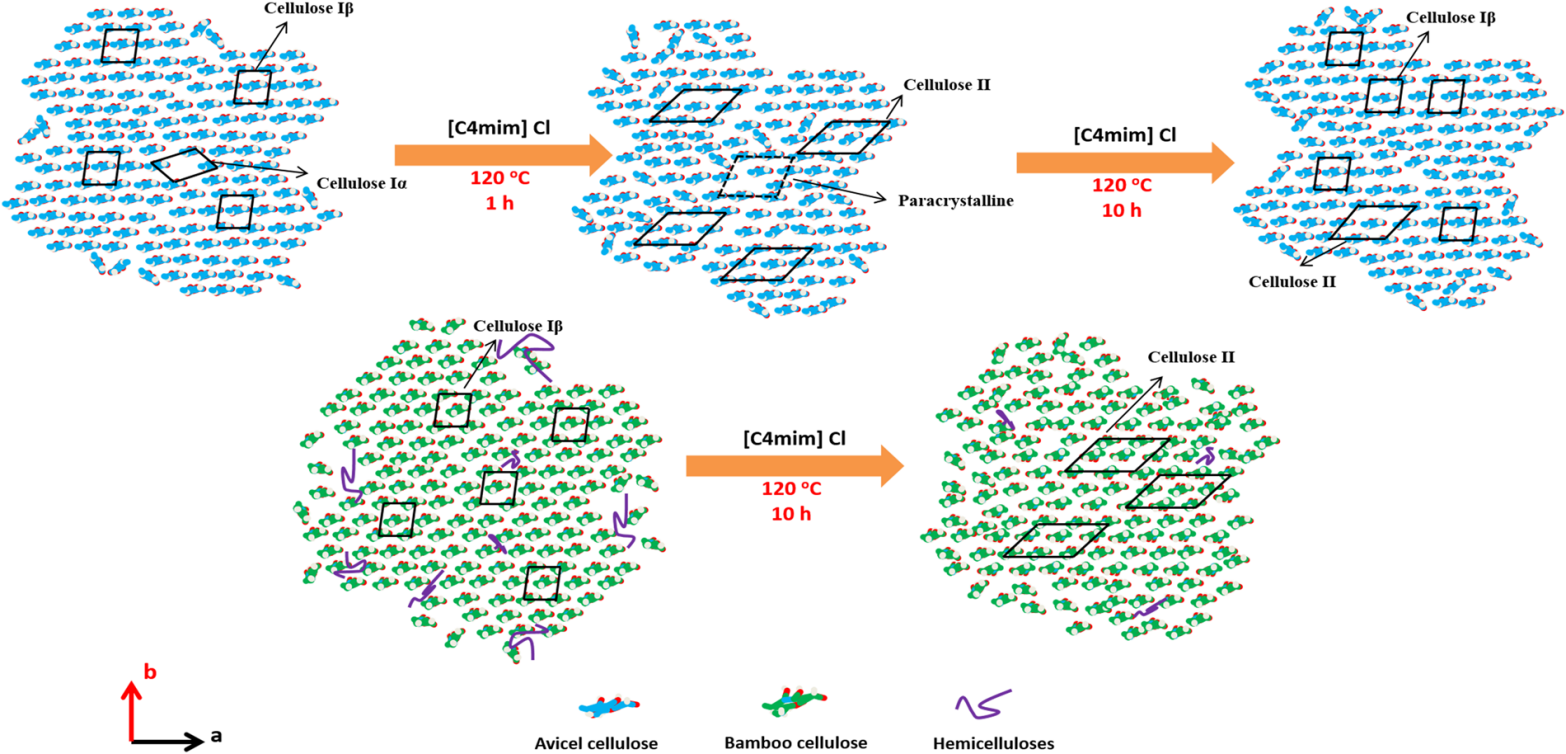
SEM images of (**a**–**d**) Avicel and (**e**–**h**) holocellulose samples (**a**,**e**) before and after IL pretreatment for (**b**,**f**) 1 h, (**c**,**g**) 4 h and (**d**,**h**) 10 h. AFM height images of (**i**) untreatedand IL pretreated holocellulose tangential sections for (**j**) 1 h, (**k**) 4 h and (**l**) 10 h.

As is known, the morphological and structural changes during the pretreatment are critical reasons contributing to enzymatic digestibility of biomass besides the chemical compositions. Field emission scanning electron microscope (FE-SEM) and atomic force microscope (AFM) images were utilized to estimate the varied surface characterizations of raw and IL pretreated substrates (Fig. 2). The structures of untreated and pretreated Avicel powders were observed (Fig. 2a–d). Untreated sample showed compactly linked microfibrils, yet the surface of Avicel powder was rough with some pores between neighbouring microfibril bundles. The prolonging pretreatment time resulted in rearrangement of microfibrils which revealed the sheet-like structures (Fig. 2b). Interestingly, after IL pretreatments for 4 and 10 h, the total realignments of fibrils appeared from regenerated samples, forming smoother surfaces than untreated Avicel, even though there were some cracks and bulges (Fig. 2c,d). The results were similar to prior studies which proposed the crystalline allomorph variations as the main causes^28^.

For untreated and pretreated holocellulose samples, powders and tangential sections were analysed respectively by SEM and AFM (Fig. 2e–l). Unlike Avicel, untreated holocellulose displayed relatively smooth and intact surface. Microfibrils were rigid and regular, embedded with hemicelluloses matrix (Fig. 2e). Collapses and cracks emerged with the increasing pretreatment time as well as some sheet-like structures, indicating that the rearrangement of microfibrils was affected by the extraction of hemicelluloses polysaccharides (Fig. 2f,g). Severe disruption of surface structure was significantly observed in Fig. 2h. Rarely surrounded by hemicelluloses, holocellulose sample for 10 h IL pretreatment demonstrated a torn fibrillar structure, which further proved complex inner transformation of cellulose microfibrils.

AFM images provided more evidential information on microfibrillar arrangements of pretreated bamboo holocellulose (Fig. 2i–l). The microfibrils arranged in order for untreated tangential section, and the fibrillar structure became more visible for 1 h pretreated sample (Fig. 2i and j). The bright spots observed on the surface were probably the incompleted dissolution of polysaccharides extruding out of fibrils. There were less bright spots in Fig. 2k (H4), which clearly exposed disordered microfibrils with some cracks around. Longer pretretment time resulted in destruction of microfibril arrangements of H10 (Fig. 2l). Meanwhile, the swelling of microfibrils was observed, as well as the shrinkage of fibril length. The phenomenon was believed to be caused from disruption of cellulose hydrogen bonding, which may affect the crystalline structures of lignocellulosic samples sensitively^29, 30^.

### Cellulose crystalline structural variations

**Figure 3:**
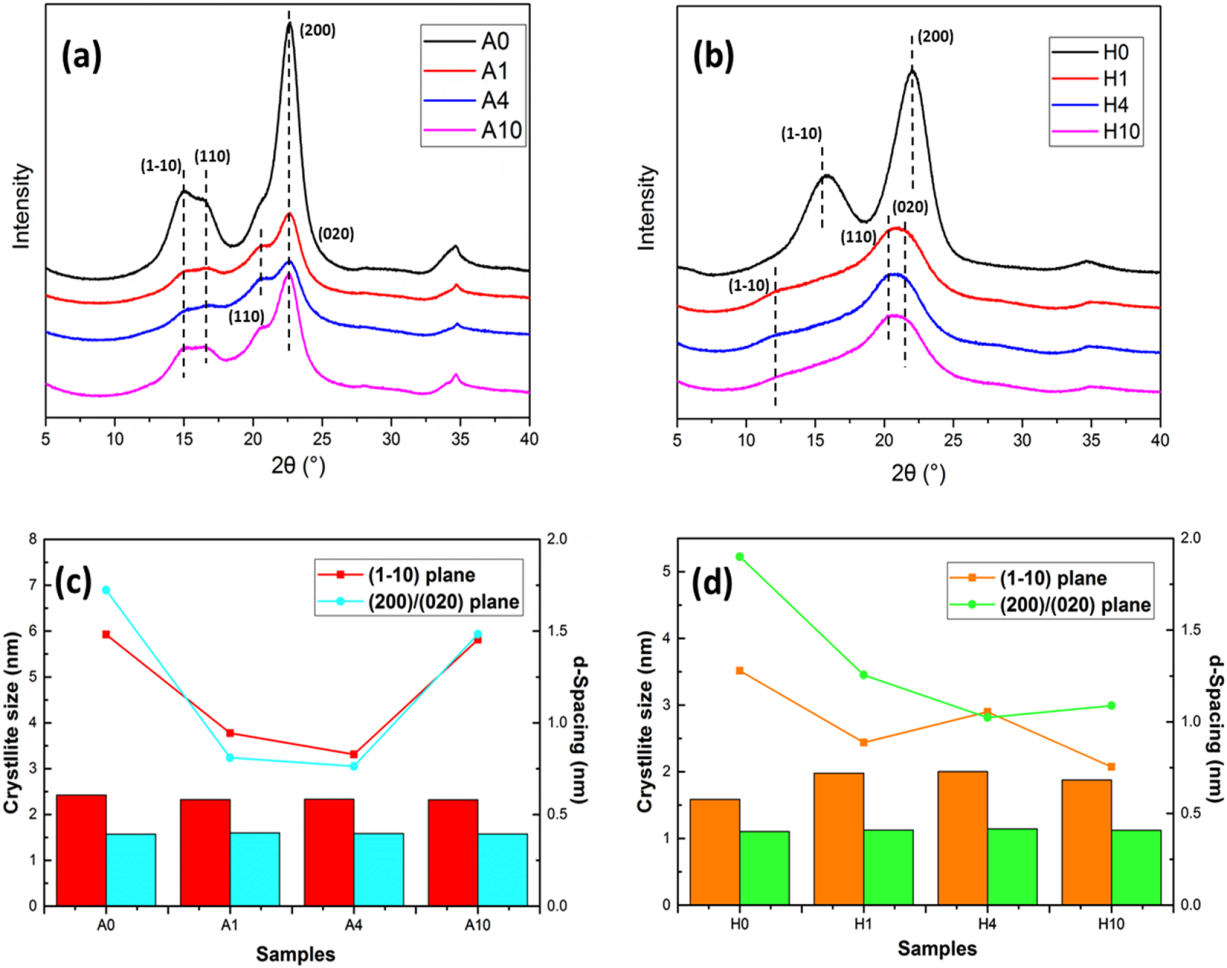
XRD diffraction patterns of (**a**) Avicel and (**b**) holocellulose with different IL pretreatment time. Crystallite sizes and *d*-Spacings of characteristic lattice plane for (**c**) Avicel and (**d**) holocellulose under different IL treatment conditions. Line chart: crystallite size; histogram: *d*-Spacing.

Considering the dramatic morphological changes of different cellulose materials during the [C4mim]Cl pretreatment, it is required to understand the mechanisms of crystalline structural changes, which is also the essential factor for bioconversion of cellulose to monosaccharides. Cellulose crystalline features during IL pretreatments were determined using XRD patterns (Fig. 3). It was observed that untreated Avicel diffraction file was identical to cellulose I, especially the peak located at 2θ = 22.5° corresponding to (200) lattice plane. The separated two broad peaks at around 15.0° and 16.8° were close to diffractions of cellulose I (14.9° and 16.7° for Iβ; 14.3° and 16.8° for Iα)^31^. With the increase of incubation time, the distinct appearance of peak at 2θ = 20.5° assigned to (110) plane of cellulose II suggested the formation of cellulose II allomorph in regenerated cellulose of A1 and A4. Interestingly, the characteristic peak for (1–10) plane of cellulose II could not be observed at 2θ = 12.5°. It may be ascribed to the remaining cellulose I allomorph and some amorphous cellulose in these two samples, which was indicated by decreased peak height at 2θ = 15.0°, 16.8° and 22.5° characteristic of cellulose I. For A1 and A4, the initial cellulose I experienced the transformation to co-existed cellulose I and II allomorph that converted some crystalline structure to amorphous phase as well^32^. It is noticed that, for 10 h IL pretreated sample (A10), the peak height at 2θ = 20.5° was reduced, whereas the reproduction of peak at 2θ = 22.5° was detected. Simultaneously, the cellulose I characteristic peak of (1–10) and (110) (2θ = 15.0°, 16.8°) was also revealed. These phenomena strongly confirmed that there was recrystallization to cellulose I for A10.

XRD patterns of holocellulose before and after IL pretreatment are displayed in Fig. 3b. Composed of hemicelluloses and cellulose, untreated holocellulse showed typical [(1–10), (200)] lattice plane at 2θ = 15.0° and 22.5° corresponding to cellulose Iβ. Prolonging pretreatment time resulted in obvious peak shift from 2θ = 15.0° and 22.5° to 12.5° and 20° that respectively reflect to (1–10) and (020) plane of cellulose II. Besides, dividing the broad peak at around 21°, it can be found the 2θ = 20.5° assigned to (1–10) plane of cellulose II, which further proved the evident conversion of cellulose I to cellulose II allomorph for holocellulose sample. In addition to the cellulose allomorph transformation, the decline of peak height with increasing time suggested some degradation of crystalline structure to amorphous region, resembling Avicel samples. However, H10 showed no recrystallization which was mainly mixed with amorphous structure and cellulose II allomorph.

Crystallinity index (CrI), crystallite sizes and crystal lattice spacings (*d*-Spacing) calculated by XRD patterns are critical factors affecting cellulose properties and digestibility to enzymes. The values of untreated and IL pretreated samples were presented in Table 2. IL pretreatment contributed to varying CrI of both samples (Table 2). Untreated Avicel predominantly containing microcrystalline cellulose had large amount of crystalline regions with high CrI (76.0%). It was observed that the CrI of A1 and A4 were 69.6% and 68.5%. The reduction indicated some destruction of crystalline areas during IL pretreatment even though cellulose II emerged in A1 and A4. Comparing CrI of A1, A4 and A10, there were no significantly continuous decrease and sample A10 still maintained high content of crystalline regions (CrI is 65.4%). It suggested that Avicel crystalline cellulose slightly degraded in ILs with short pretreatment time (1 h) and few amount of amorphous structures generated afterwards, in addition to some inner crystalline transformations. With more amorphous regions, the CrI for untreated holocellulose (A0) was 47.6%, much lower than Avicel (76.0%), much lower than A0. IL pretreatment resulted in remarkable decline of CrI to 28.4% of H1, which constantly reduced to 17.6% (H4) and 11.2% (H10). It was noticed from XRD that antiparallel chains of cellulose II dominated in H1, H4 and H10 samples. ILs seemed to be easier to penetrate in biomass samples than highly crystallized Avicel, further causing less crystalline regions in pretreated holocellulose.

**Table 2:** Crystallite sizes, *d*-Spacings and crystallinity indices of Avicel and moso bamboo holocelluloses after ionic liquid pretreatment.

Samples	Lattice plane	A0	A1	A4	A10	H0	H1	H4	H10
CrI^c^ (%)	—	76.0	69.6	68.5	65.4	47.6	28.4	17.6	11.2
L^a^	(1–10)	5.9247	3.7748	3.3115	5.8129	3.5150	2.4408	2.8984	2.0725
(nm)	(110)	3.2889	3.1854	2.8452	3.1466	4.3853	2.2377	1.9351	1.7397
	(200)^d^	6.8945	3.2373	—	5.9321	5.2257	—	—	—
	(020)^e^	—	—	3.0492	—	—	3.4514	2.8141	2.9915
*d*-Spacing^b^	(1–10)	0.6057	0.5808	0.5828	0.5800	0.5765	0.7189	0.7281	0.6820
(nm)	(110)	0.5402	0.5195	0.5287	0.5290	0.4998	0.4298	0.5043	0.4318
	(200)	0.3923	0.3996	—	0.3939	0.4012	—	—	—
	(020)	—	—	0.3959	—	—	0.4092	0.4164	0.4080

^a^Crystallite size perpendicular to the direction of each plane.

^b^ *d*-Spacing of three equatorial peaks of cellulose in Fig. 3.

^c^Crystallinity index.

^d^(200) plane of cellulose I.

^e^(020) plane of cellulose II.

Figure 3c,d demonstrated no obvious changes distance between different lattice planes except for slightly increased (1–10) *d*-Spacing of H1, H4, H10 than untreated holocellulose (H0). Less hemicelluloses were coalescent within hydrophobic plane of cellulose chains and intercalation of IL molecules. Moreover, the spartial constraints impeded the approach of cellulose crystals in ILs^33^. Untreated Avicel and holocellulose were composed of cellulose I allomorph with high crystalline structures. The crystallite sizes of [(1–10), (110), (200)] plane were respectively 5.9247 nm, 3.2889 nm, 6.8945 nm for A0 and 3.5150 nm, 4.3853 nm, 5.2257 nm for H0. With the increasing of IL pretreatment time, crystallite sizes reduced along with the (200) plane converting to (020) plane of cellulose II in A4, H1, H4, H10 revealed by XRD. For A10, which was mainly recrystallized cellulose I dominated Avicel sample, the crystallite sizes were 5.8129 nm, 3.1466 nm and 5.9321 nm for [(1–10), (110), (200)] lattice planes (Table 2, Fig. 3d). The boosted crystallite sizes furtherly proved the rearrangement of crystals and recrystallization to a more ordered crystalline structure.

### Specific crystalline analysis by Nuclear magnetic resonance (NMR) and Z-plot

**Figure 4:**
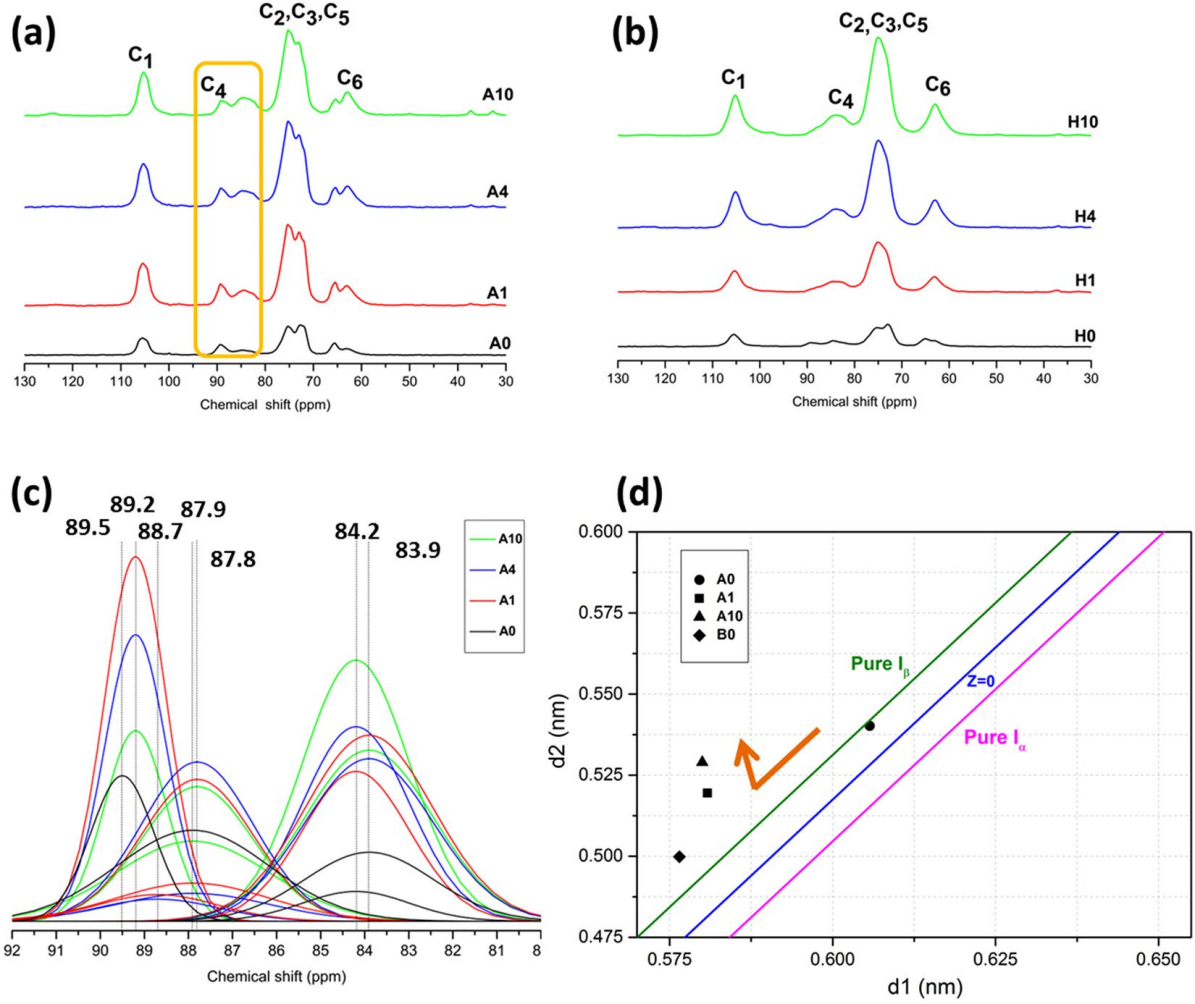
Solid state ^13^C CP-MAS NMR spectra of untreated and ILs pretreated (**a**) Avicel and (**b**) holocellulose sample. (**c**) enlargement for fitted C4 region of ILs pretreated Avicel; (**d**) Z plot of the cellulose I dominated samples (A0, A1, A10 and H0). The Z = 0 boundary line exists between Iα rich and Iβ dominant types, representing the varying Iα/Iβ ratios.

^13^C CP-MAS NMR spectra of Avicel and holocellulose depicted more explicit information for supramolecular structural and chemical variations during ILs pretreatment (Fig. 4). Initial cellulose sample presented typical resonance peaks from 60 ppm to 110 ppm (Fig. 4a). The resonance at 106.0 ppm and 65.6 ppm of Avicel sample corresponded to the C1 and C6 of cellulose I lattice. Broad peak at 80–90 ppm was assigned to C4 resonance, which provided abundant details about crystalline features^34^. Diverse crystalline structures and polymers residing at fibril surfaces of ILs pretreated Avicel were displayed by peak deconvolution of C4 region (Fig. 4c). For untreated Avicel (A0), two distinct peaks at 89.2 ppm and 87.9 ppm respectively ascribed to cellulose Iα and Iβ confirmed the mixture of these two polymorphs in A0. The result was in accordance to XRD patterns. ILs pretreatment contributed to the disappearance of 89.2 ppm for A1 and A4, whereas there exhibited slight peak at 88.7 ppm due to paracrystalline resonance, as well as obvious peak at 89.2 ppm and 87.8 ppm due to C4 resonance for cellulose II^35^. Furthermore, increase of 63.9 ppm corresponding to cellulose II of C6 resonance also indicated the conversion to cellulose II allomorph after ILs generation (Fig. 4a). Peak fitting results in Fig. 4c proved the recrystallization behaviour of Avicel at 87.9 ppm, where the cellulose Iβ resonance increased for A10 compared to A1 and A4. Simultaneously, increased amorphous regions of Avicel samples were revealed by higher resonance peak at around 83 ppm^36^. Deconvolution of this region also presented detailed assignments of accessible and inaccessible fibril surfaces at 84.2 ppm and 83.9 ppm (Fig. 4c). Compared to untreated sample (A0), both two peaks became sharper and stronger after IL pretreatment, indicating that the swelling of microfibrils caused conformation between surface and interior polymers^34^. Samples A1 and A4 showed no clear differences between accessible and inaccessible fibril surfaces, while it was visible that A10 had increased accessible surface area. It may be attributed to more ordered crystallites of recrystallized cellulose I, which exposed more microfibril chains to the surfaces.

Crystalline structural and chemical variations of holocellulose samples were also identified (Fig. 4b). For sample H0, a broad peak was observed at around 73.2 ppm originate from hemicelluloses linkages with neighboring peak for C5 resonance in glucan at 74.8 ppm^25, 37^. The weaker peak in this resonance region suggested the extraction of polysaccharides, corresponding to compositional analysis and FTIR results. Similarly, for C6 region of holocellulose sample, the peak at 66.6 ppm (H0) assigned to C5 in β-D-xylan units disappeared with the prolonging pretreatment time. Besides, the increasing peak of 63.9 ppm in this region was due to the stronger C6 resonance of cellulose II, reflecting the cellulose allomorph transformation. The crystalline variation was also revealed for C1 region, where the peak of C1 resonance shifted to 105.7 ppm assigned to regenerated cellulose II^38^

As mentioned above, the cellulose crystalline alternations of Avicel and holocellulose during IL pretreatment were not necessarily accordant. Especially, quantitative ratio of allomorph domains of IL pretreated Avicel has not been fully elucidated. According to Wada *et al*. (1993), the *d*-Spacings of (1–10), (110), (200) lattice plane for cellulose crystal were marked as d_1_, d_2_ and d_3_ respectively^39^. The d_1_ and d_2_ value in Table 2 can be jointly used to determine the ratio of cellulose Iα and Iβ by the function:

$${\rm{Z}}={{\rm{1693d}}}_{1}-{{\rm{902d}}}_{2}-549$$

where Z > 0 for the cellulose Iα type and Z < 0 for the cellulose Iβ type (Table 3). Z values of cellulose I allomorph samples were calculated as negative, indicating the mainly monoclinic chains (cellulose Iβ) of untreated Avicel and holocellulose. The values of Avicel samples decreased with rising pretreatment time, expect for undefined A10, in which cellulose II occupied a large content. The phenomenon suggested that, the recrystallization behaviour found by XRD analysis caused the generation of more cellulose Iβ domains.

**Table 3:** Z-values of cellulose I dominated samples (A0, A1, A4 and H0) calculated via *d*-Spacings of (1–10) and (110) lattice plane.

Sample	d1 (nm)	d2 (nm)	Z value
A0	0.6057	0.5402	−10.8103
A1	0.5808	0.5195	−34.2946
A4	0.5800	0.5290	−44.2180
H0	0.5765	0.4998	−23.8051

Additionally, the actual Iα/Iβ ratio was calculated by measuring and plotting d_1_ and d_2_ (Fig. 4d)^40^. As estimated, location of d1/d2 for Avicel (A0) right of boundary for cellulose Iβ, showing small initial amount of cellulose Iα in Avicel sample. The results resembled the former researches on cotton and wood pulp^41^. The coordinates of A1 and A10 were dramatically left shifted, where pure cellulose Iβ dominated the sample after IL pretreatment. It confirmed the conversion of original cellulose Iα to cellulose Iβ during recrystallization under (C4mim)Cl pretreatment. The results were perfectly coincident with FTIR spectra of Avicel samples (Fig. 1). As prolonging pretreatment time, the peak 710 cm^−1^ attributed to Iβ cellulose markedly raised together with sharpened band of 3240 cm^−1^ ascribed to cellulose Iα, which also suggested the inner recrystallization to cellulose Iβ^42, 43^.

**Figure 5:**
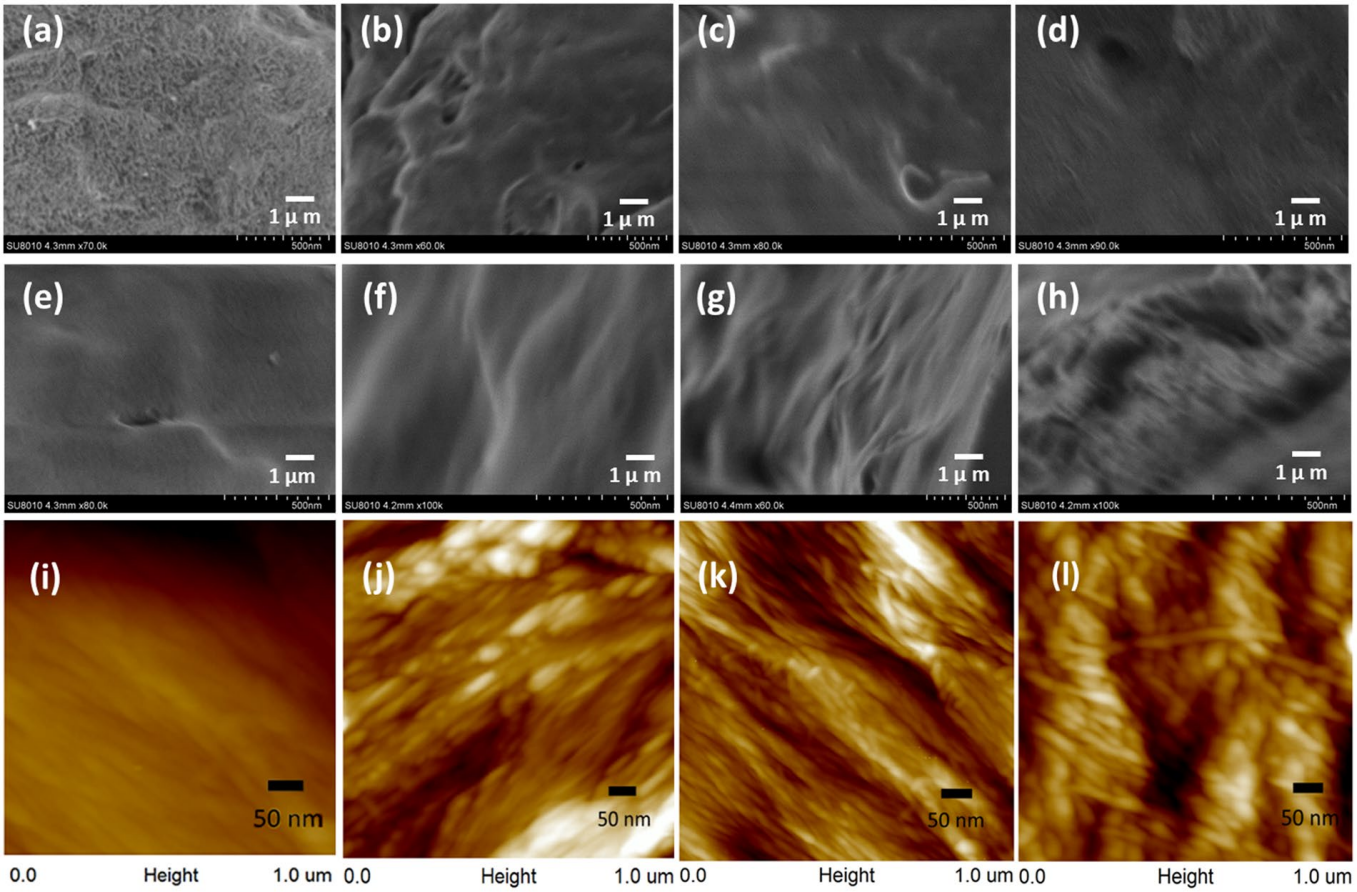
Schematic representation of IL pretreatment induced crystallite transformations for the microfibril cross-section of Avicel and holocellulose.

Considering the slightly reduced crystallinity and partial transformation to cellulose II, we can hypothesize that, the process of Avicel recrystallization was completed by forming the intermediate paracrystalline phases upon interaction of IL with cellulose chains (Fig. 5). Intermediate structure generated in biomass samples and lignin were reported to restrain the transformation to cellulose II during [C2mim][OAc] pretreatment^17, 33^. As a result, the recrystallization behaviour and generation of intermediate phases may prohibit the converting to cellulose II allomorph of Avicel, which is totally different from the transformation rules of IL pretreated holocellulose.

### Enzymatic hydrolysis

**Figure 6:**
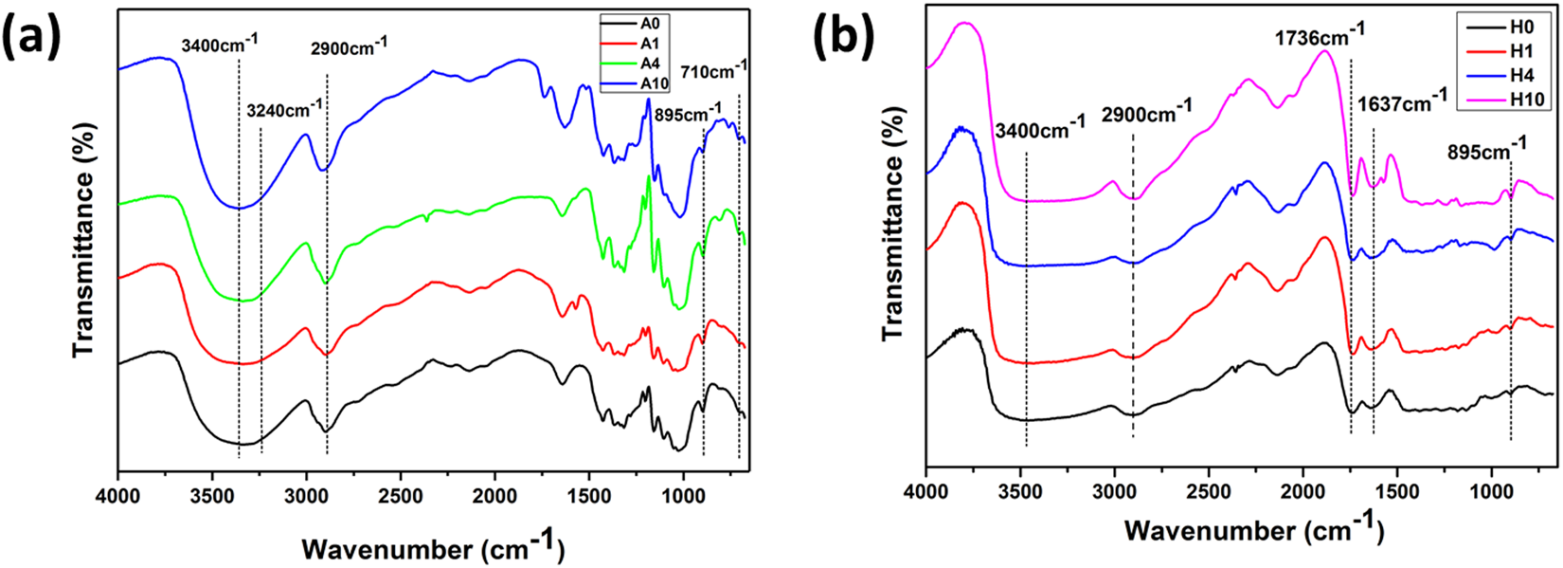
(**a**) Cellulose conversion yield of Avicel and holocellulose samples with different IL pretreatment time. (**b**) comparisons of cellulose crystallinity and glucose yield after 72 h incubation. Error bars indicated standard deviation of triplicate determinations.

The performance of enzymatic hydrolysis was evaluated by the conversion yield of glucose from cellulose substrates (Fig. 6a). In this study, enzymatic saccharification assays were conducted under the same conditions to ensure the validity of comparison^44^. For Avicel samples, IL pretreatment demonstrated no stimulating effects on enzymatic hydrolysis. After 72 h incubation with enzymes, the glucose yield of A0 was 51.5%, whereas the value experienced slight reduction to 44.71% (A1) and 35.68% (A4). Similarly, the conversion rate of A1 and A4 were slow. Sample A10 represented a little higher glucose yield (37.42%) than A4, while it still remained at a low level. The CrI of Avicel and the glucose yield after 72 h hydrolysis were compared (Fig. 6b). CrI of Avicel decreased from 0 h (A0) to 4 h (A4) treatment, together with allomorph transformation from cellulose I to cellulose II. The process contributed to rotation of cellulose chains, which may affect the digestibility of cellulose to enzymes. For cellulose II dominated samples, the conversion yield was reported to be affected positively by CrI, indicating that lower CrI may result in less glucose from enzymatic hydrolysis^24^. Besides the existence of cellulose II, the formation of intermediate paracrystalline from cellulose Iα held together more hydrogen bonding and van der Waals forces, restricting penetration of enzymes^45^. A10 showed typical crystalline characterization of cellulose Iβ, in which the CrI acted as the obstacle for glucose conversion^46^. Slight decline of CrI that suggested more amorphous regions in A10 broke some recalcitrance of crystalline cellulose, inducing higher enzymatic digestibility of cellulose than A4. However, the recrystallization behaviour of A10 resulted in typical crystalline allomorph of cellulose Iβ, which became a main reason for still low glucose conversion yield (37.42%).

Biomass samples seemed more prone to be affected by IL pretreatment. The conversion yield of untreated holocellulose to glucose after 72 h was 66.78%, which was nearly constant since hydrolysed with 16 h. The sample displayed higher hydrolysis rate and conversion yield compared to both Avicel and raw bamboo materials detected by previous researchers^19^. It can be explained by lower cellulose crystallinity and no lignin matrix hindering the enzymatic accessibility. As expected, the conversion yield of IL pretreated holocellulose boosted to 90.70%, 88.68% and 92.20% for H1, H4 and H10, apparently higher than those of IL pretreated Avicel. Presenting similar conversion rate in the first 5 h as untreated holocellulose, H1, H4 and H10 released more glucose after hydrolysis for 16 h. The constant dissolution of hemicelluloses that weakened the hydrogen linkages of polysaccharides played an important role in improving the enzymatic accessibility to cellulose. Taking the CrI into consideration, the demonstrable reduction of H1 CrI reflected the massive generation of amorphous cellulose, facilitating the biomass more susceptible to enzymes (Fig. 6b). Furthermore, XRD implied cellulose II as the main crystalline allomorph for H1, which may provide easier digestibility to cellulase than cellulose I^47^. The rise of cellulose conversion yield was retarded for H4 and H10, showing scarce variation. It was ascribe to the accomplishment of cellulose crystalline alternation, making H4 and H10 remained with full mixture of cellulose II and amorphous cellulose.

## Conclusions

[C4mim]Cl induced varying crystalline transformation on different cellulose samples. Recrystallization of cellulose Iβ was detected for 10 h pretreated Avicel, which was mainly originated from small amount of original cellulose Iα via formation of intermediate paracrystalline phase. Initially high crystalline order and the recrystallization behaviour during IL pretreatment maintained the high crystallinity of Avicel, hindering the following enzymatic conversion to glucose. For IL pretreated holocellulose, enlarged distance of lattice planes, reduction of both crystallinity and crystallite size were revealed, which jointly promoted the enzymatic digestibility. This study may contribute to innovative interpretations for crystalline variations of IL pretreated cellulose samples, thus developing the application of IL pretreatment for enhanced bioconversion to monosaccharides.

## Materials and Methods

### Materials

Two kinds of cellulose materials were applied in this study. Avicel PH101, purchased from Sinopharm Chemical Reagent Co. Ltd, Shanghai, China, is a kind of microcrystalline cellulose containing more than 97% cellulose and has highly ordered structure. Holocellulose was extracted from moso bamboo (*Phyllostachys edulis*) (provided by Forestry Center of Lishui, Zhejiang, China). Bamboo material was milled with a FZ120 plant shredder (Truelab, Shanghai, China) and passed through an 80-mesh screen. After an exhaustive extraction with toluene/ethanol (2:1, v/v) by a Blst-250SQ Soxhlet apparatus and oven drying, the samples were treated with sodium chlorite/acetic acid at 75 °C for up to 4 h to remove the impact of lignin. The lignin-free samples (holocellulose) were washed by deionized water extensively until a neutral pH was reached, followed by oven-drying at 60 °C for 12 h.

### IL pretreatment

The 1-butyl-3-methylimidazolium chloride, [C4mim]^+^Cl^−^ with stated purity ≥ 98.5%, was purchased from Lanzhou Institute of Chemical Physics of the Chinese Academy of Sciences (Lanzhou, China). The 5 g of solid powder (Avicel or holocellulose) was mixed with 95 g of [C4mim]Cl in a 250 mL dried three-neck flask with stirring at 100 rpm under nitrogen atmosphere. The isothermal pretreatments were performed at 120 °C with varied treating time (1 h, 4 h and 10 h). Untreated Avicel and holocellulose are marked as A0 and H0, and samples with different treating time are marked as A1, A4, A10 and H1, H4, H10. After the reaction was completed, the samples were respectively transferred to 300 mL of deionized water to precipitate the biomass. The mixture of IL, water, biomass was then centrifuged to separate the liquid (IL and water) and solid (regenerated biomass) phases. The recovered solids were washed thoroughly with hot water for 6 times and freeze-dried for 2 days. The pretreatment processing was performed in triplicate.

### Enzymatic hydrolysis

The native and pretreated samples (Avicel and holocellulose) were enzymatically hydrolysed in a 0.05 M sodium acetate buffer with a pH of 4.8 at a biomass loading of 10% (w/v) in an air-shaking incubator maintained at 48 °C and 150 rpm for 72 h. The cellulase used in this work was provided by Shanghai Youtell Biochemical Ltd. (Shanghai, China) and was a mixure of endo-β-(1,4)-glucanace, exo-β-(1,4)-D-glucanase and β-D-glucocidase extracted from submerged fermentation of *Trichoderma*. Cellulase was employed at an activity of 20 FPU/g. The enzymatic reactions were controlled by taking 100 μL supernatant at specific time intervals, followed by deactivation and centrifugation. The released monosaccharides were analyzed by high-performance anion exchange chromatography (HPAEC) system (ICS-3000, Dionex, California, USA). The hydrolysis processing was conducted in triplicate.

### Chemical components analysis

The determination of structural carbohydrates of pretreated solid samples was estimated according to the National Renewable Energy Laboratory of the U.S. (NREL) analytical methods for biomass^48^. The sugar content was determined after a two-step acid hydrolysis procedure. The powder samples were treated with 72% (w/w) H_2_SO_4_ at 30 °C for 1 h in the first step. The reaction mixture was then diluted to 4% (w/w) H_2_SO_4_ and autoclaved at 121 °C for 1 h. The hydrolysis solution was filtered and analyzed for sugar content by HPAEC under the same condition as enzymatic hydrolysis.

### X-ray diffraction

X-ray powder diffraction patterns of untreated (A0, H0) and treated samples (A1, A4, A10, H1, H4, H10) were obtained by a Bruker D8-Advance instrument (Bruker, Germany). Samples were scanned from 2θ = 5° to 2θ = 40° with increments of 0.02° using the Cu Kα radiation (λ = 1.54 Å). The data of XRD were stored and calculated by software MDI Jade 5.0.

CrI was determined by means of following equation via the height of the 200 peak (*I*_200_, 2θ = 22.5°) and the minimum between the 200 and 100 peaks (*I*_am_, 2θ = 18°)^49^.

1 $$CrI=\frac{({I}_{200}-{I}_{am})}{{I}_{200}}\,$$

The crystal size (dimension) *L*_*hkl*_ was calculated according to the formula:

2 $${L}_{hkl}=\frac{0.9\lambda }{{B}_{hkl}\,\cos \,\theta }$$

where *λ* is the X-ray wavelength; *B*_*hkl*_ is the angular full-width at half maximum intensity (FWHM) in radians of the (*hkl*) line profile; *θ* is the scattering angle^11^.

The lattice spacing *d*_*hkl*_ was determined for each profile from the resolved position *Xc* and by using the Bragg equation (Equation 3) (Klug and Alexander, 1954)^50^:

3 $$n\lambda =2{d}_{hkl}\,\sin \,\theta $$

where *n* is an integer; *λ* is the wave length of the X-rays, *θ* is the scattering angle; and *d*_*hkl*_ is the distance between the atomic layers (lattice spacing).

### FTIR and NMR Spectroscopy

The effects of IL pretreatment on the chemical compositions were studied using FTIR spectroscopy. The air-dried samples were placed in a Thermo Scientific Nicolet iN10 Fourier transform infrared spectrometer (USA). The IR spectra were recorded in the wave number range of 700 to 4000 cm^−1^ at 4 cm^−1^ resolution for 20 scans prior to the Fourier transformation. All peak intensities were recorded and analyzed by FTIR software.

NMR measurements were conducted by a ^13^C CP-MAS NMR spectrometer (Bruker AVANCE-III AV 400 MHz, Germany) operating at 100.6 MHz. All samples put in a 5-mL centrifuge tube before being sent to NMR measurements. The chemical shifts were determined in CP-MAS (Cross-polarization magic angle spinning) spectra using the cellulose C-1 signal as an internal standard (δ105 ppm).

### FE-SEM and AFM

FE-SEM analysis was conducted using a Hitachi S-3400 N scanning electron microscope operated at 10 kV acceleration voltages. All the samples were spread on a metalcylinder plate with a carbon tape and coated with gold prior to examination.

Bamboo chips were cut by a Thermo slide microtomes into tangential sections (thickness is 30 μm), followed by the delignification and IL pretreatment under the equal condition as powders. The surface topography of untreated and pretreated holocellulose sections were measured using a Nanoscope V Multimode eight atomic force microscope (AFM) (Bruker, Germany). The height images were collected in ScanAsyst mode in air at room temperature with a silicon tip. The software Nanoscope Analysis was used for image processing.
